# Molecular Mechanisms of Immune Regulation: A Review

**DOI:** 10.3390/cells14040283

**Published:** 2025-02-14

**Authors:** Borros Arneth

**Affiliations:** 1 Institute of Laboratory Medicine and Pathobiochemistry, Hospital of the Universities of Giessen and Marburg UKGM, Philipps University Marburg, Baldingerst 1, 35043 Marburg, Germany; borros.arneth@staff.uni-marburg.de; 2 Institute of Laboratory Medicine and Pathobiochemistry, Hospital of the Universities of Giessen and Marburg UKGM, Justus Liebig University Giessen, Feulgenstr. 12, 35392 Giessen, Germany

**Keywords:** immune checkpoints, cytokine signaling, epigenetic modifications, non-coding RNAs, regulatory T cells (Tregs)

## Abstract

Background: The immune system must carefully balance fighting pathogens with minimization of inflammation and avoidance of autoimmune responses. Over the past ten years, researchers have extensively studied the mechanisms regulating this delicate balance. Comprehending these mechanisms is essential for developing treatments for inflammatory conditions. Aim: This review aims to synthesize knowledge of immunoregulatory processes published from 2014–2024 and to highlight discoveries that provide fresh perspectives on this complex balance. Methods: The keywords “molecular mechanisms”, “immune regulation”, “immune signaling pathways”, and “immune homeostasis” were used to search PubMed for articles published between 2014 and 2024, with a preference for articles published in the past three years. Results: Recent research has pinpointed the impact of factors such as cytokine signaling, T-cell regulation, epigenetic regulation, and immunometabolism on immune function. Discussion: New research highlights the intricate interactions between the immune system and other molecular elements. A key area of interest is the impact of non-coding RNAs and metabolic pathways on the regulation of immune responses. Conclusions: Exploring the mechanisms by which the immune system is regulated will provide new avenues for developing treatments to address autoimmune and inflammatory conditions.

## 1. Introduction

The immune system has to strike a careful balance between tolerance to self-antigens and defense against infections. Precise molecular mechanisms that control immune activation and avoid excessive or inappropriate immunological responses are responsible for achieving this equilibrium. The topic of immune regulation is broad and complex, involving many different processes, including sirtuins, autophagy, inflammasome activation, mitochondrial function, post-translational changes, miRNA-mediated epigenetic control, and TLR signaling. Because immune regulation is so broad, it is not possible to adequately examine every facet in a single paper. Therefore, the main processes that are essential for preserving immunological homeostasis are the subject of this review. These mechanisms include immunometabolism, cytokine signaling, immune checkpoints, regulatory T cells, and epigenetic changes.

Research on these regulatory systems has evolved significantly over the past ten years, exposing the intricate interactions among metabolic and epigenetic changes, cellular checkpoints, and signaling pathways. Although these mechanisms are intricately interrelated, many parts are still poorly understood. In order to present a current analysis of the molecular mechanisms of immune regulation, this systematic review integrates findings from the literature published between 2014 and 2024. It highlights new findings, their interactions, and their potential therapeutic applications in the treatment of autoimmune diseases, inflammatory conditions, and immune-related disorders.

## 2. Historical Perspectives on Immune Regulation

The control of the immune system has been an area of immense interest among scientists for nearly half a century. Research has traced some of the roots of modern understanding of immunity to very few initial observations. The main landmarks in this field comprise identifying antibodies, acknowledging their specificity, and discovering lymphocytes and their capacity for immune reactions [1]. The first discoveries in immune regulation included tolerance and autoimmunity, some early theories being Burnet’s clonal selection theory in the 1950s, which explained the immune system’s self-recognition [2]. These formed the basis for further investigations into how an immune response is initiated, regulated, and terminated.

Nevertheless, several questions remain unanswered despite these advancements. For instance, how is tolerance in terms of immunity sustained when there are intricacies in microenvironments, and what molecular markers guarantee the stability of the regulatory systems? Altogether, these questions are evidence of the continuous need to investigate the fundamental mechanisms of immune regulation further.

### Deeper Historical and Mechanistic Analysis

Tregs were discovered in the 1990s and are a specialized subset of a cluster of differentiation 4 (CD4) T cells recognized to be constitutionally pivotal for immunologic tolerance and autoimmunity suppression [3]. Established through the T-cell-specific transcription factor, FOXP3, Tregs use pathways such as cytokines, metabolites, and cell contacts to modulate immune responses [3]. Interleukin-10 appeared on the scene in the late 1990s, and this cytokine was found to inhibit the synthesizing and promoting of proinflammatory cytokines, mostly in macrophages and dendritic cells, thereby preventing further tissue injury [4]. Likewise, the discovery further advanced regulation in the early 2000s by providing a novel layer of immune regulation through maintaining characterization as regulatory non-coding RNA that controls target mRNA stability and translation.

Functionally, FOXP3 modulates Treg function regarding the secretions of the immunosuppressive cytokines such as Interleukin (IL-10) and transforming growth factor beta (TGF-β), which are important in immune tolerance [3]. Protein IL-10 interacts with its receptor expressed on target cells through binding, activating the Janus kinase/signal transducers and activators of transcription (JAK/STAT) signal transduction pathway to control inflammation response [5]. miRNAs, including miR-146 and miR-155 have important regulation functions [4], in which miR-146 reduces NF-κB signaling through targeting Interleukin-1 receptor-associated kinase (IRAKI) and tumor necrosis factor receptor-associated factor 6 (TRAF6) [6,7], and miR155 affects cytokine production through regulation of Suppressor of Cytokine Signaling 1 (SOCS1) level [8,9]. Other anti-miRs, including miR-21 and miR-223, control immune reactions related to T-cell activation and macrophage polarization, correspondingly [10,11]. These mechanisms demonstrate how miRNAs modulate immune responses by integrating their regulatory role throughout all immune cell types.

Nevertheless, there remain many questions unanswered. For instance, since the discovery of miRNAs, the complex interaction between these molecules and other regulating molecules in various immune scenarios has yet to be fully established. Furthermore, the contribution of other recently discovered non-coding molecules, including lncRNAs, in the regulation of the immune system remains largely unknown, indicating potential future development in immune-related research. Knowledge of such mechanisms is vital to the formulation of new approaches to treating diseases related to immune dysregulation.

## 3. Recent Developments in Immune Regulation

In recent decades, considerable advances have been made in previously unresolved questions. Breakthroughs such as identifying definite regulatory molecules, including the transcription factor forkhead box P3 (FOXP3) in Tregs and microRNAs, have highlighted the complex pathways used to control the immune system [3]. For instance, FOX transcription factors, such as FOXP3, are essential for Treg development and function and are important for immune regulation [3]. Likewise, microRNAs (miRNAs) and other non-coding RNAs have been identified as exerting critical control of cytokine signaling and differentiation of immune cells. Examples of such specific miRNAs that have been reported to directly modulate the immune system and thus influence health and disease include miR 146a and miR 155 [4].

The discovery of immunometabolism has greatly revolutionized this category. Recent discoveries of key molecules such as FOXP3 in Tregs and miRNAs in the past decade have highlighted the complexity of the immunosuppressive process [3]. For example, glycolysis sustains the high energy needs of effector T cells during an immune response, while oxidative phosphorylation is important for Tregs’ suppressing function [7]. Their metabolic preferences thus illustrate dynamically the interdependence of cellular energetic states with immune regulation.

The rapid advancement in single-cell technologies and high-throughput sequencing has accelerated further discoveries in this area. Such tools allow researchers to dissect the heterogeneity of immune cell populations and uncover novel regulatory molecules for new perspectives on immune regulation with unprecedented resolution. Such innovations enhance our understanding and provide promising avenues for precision medicine in immune-related disorders.

## 4. Technical and Philosophical Impacts

These recent findings have profound technical and philosophical implications for immunology. Technically, these findings add new molecular targets for therapeutic intervention, especially for diseases related to immune dysregulation. Such instances are found in autoimmune conditions, chronic inflammatory disorders, and even the hyperinflammatory state observed in severe cases of COVID-19 cytokine storms, which call for much-needed novel approaches for immunomodulation.

In a philosophical manner, the expanded understanding of immune regulation challenges previous views of immunity as a binary system of defense, underlining the intricacy of immune tolerance and activation as interdependent processes requiring sensitive molecular orchestration. This notion increases our appreciation of the intricacies within the immune system and points toward the need for a systems approach in studying and manipulating immune responses.

## 5. Description of the Immune System: Composition and Function

The immune system is a sophisticated network that keeps the body self-tolerant while protecting it against infections. Adaptive immunity and innate immunity are its two primary branches. Innate immunity offers primary, non-specific protection through physical barriers (such as mucosa and skin), soluble molecules (including cytokines and complement proteins), and immune cells (such as neutrophils, macrophages, and innate killer cells). In particular, innate immunity acts as the first line of protection and bridges adaptive immunity via the presentation of antigens by dendritic cells.

Adaptive immunity grows over time and is quite particular. It involves T cells, such as cytotoxic T cells (which target infected cells) and helper T cells (which coordinate responses), as well as B cells (which produce antibodies). Regulatory T cells (Tregs) are the aspects of CD4+, and form the central part of the immune regulation; Tregs are crucial for preventing autoimmunity by reducing the intensity of immune responses [12].

Important molecules like chemokines, cytokines, and co-stimulatory signals bridge communication between adaptive and innate immunity, establishing a balance in immune response. Modulating mechanisms such as regulatory T cells bring about tolerance and homeostasis, closely aligning to the focus of my research.

## 6. Mechanisms of Immunological Modulation and an Explanation of the Processes

By striking a balance between immune activation and suppression, immune regulatory systems preserve homeostasis and avert autoimmune disorders and excessive inflammation. Important mechanisms include sirtuins, autophagy, inflammasome activation, mitochondrial function, post-translational changes, Toll-like receptor (TLR) signaling, and microRNAs (miRNAs) in epigenetics [13,14,15,16].

To avoid overreactions from the immune system, autophagy breaks down infections and damaged organelles. Multiprotein complexes known as inflammasomes alter innate immunity by activating inflammatory cytokines such as IL-1β. Energy metabolism and T-cell activation are influenced by mitochondrial activity, whereas sirtuins, which are NAD+-dependent enzymes, control inflammation and stress reactions. Immune signaling pathways are refined by post-translational changes like phosphorylation and ubiquitination.

In epigenetics, miRNAs influence immune cell differentiation by suppressing mRNA, which regulates gene expression. When pathogens are detected by TLR signaling, adaptive immune responses and cytokine production are triggered. Together, these systems guarantee tissue healing, pathogen protection, and immunological tolerance. Autoimmunity, metabolic diseases, and chronic inflammation are caused by dysregulation, underscoring the necessity of precise immunological modulation [17].

### 6.1. Immune Regulatory Processes

#### 6.1.1. Autophagy

Autophagy is a process of cellular breakdown that maintains immunological homeostasis by eliminating damaged proteins and organelles. It controls inflammation, pathogen removal, and antigen presentation. Chronic inflammation, autoimmune disorders, and compromised immune surveillance in cancer and infections are all impacted by autophagy dysfunction [18,19,20,21].

#### 6.1.2. Inflammasome Activation

Multiprotein complexes called inflammasomes identify threats and infections, triggering caspase-1 to release IL-1β and IL-18. Inflammatory reactions are triggered by this. Dysregulated inflammasome activity affects tissue homeostasis and innate immune defense, which in turn leads to chronic inflammation, metabolic problems, and autoimmune illnesses [21].

#### 6.1.3. Mitochondrial Function

Reactive oxygen species (ROS) and ATP produced by mitochondria control immunological responses by affecting immune cell activation, differentiation, and death. Mitochondria dysfunction impairs pathogen defense, increases autoimmunity, and causes inflammation. Additionally, they are crucial for immune cell metabolic reprogramming [22].

#### 6.1.4. Role of Sirtuins

NAD+-dependent deacetylases known as sirtuins control immunological function by means of longevity, inflammatory suppression, and metabolic adaptability. They alter the polarization of macrophages, the generation of cytokines, and T-cell differentiation. Autoimmune diseases, age-related immunological dysfunction, and chronic inflammation are all associated with sirtuin dysregulation [22].

#### 6.1.5. Post-Translational Modifications

PTMs, such as acetylation, phosphorylation, and ubiquitination, adjust protein stability, location, and activity to fine-tune immunological signals. They control immunological tolerance, antigen presentation, and cytokine signaling. Defective pathogen responses, inflammatory illnesses, and immune evasion in cancer are all influenced by aberrant PTMs [23,24,25].

#### 6.1.6. MicroRNAs in Epigenetics and TLR Signaling

By directing mRNAs towards translational repression or destruction, microRNAs (miRNAs) control the expression of immunological genes. They shape innate and adaptive immunity by influencing TLR signaling and epigenetic changes. By affecting immunological homeostasis, dysregulated miRNAs play a role in cancer, infections, and autoimmune disorders [26,27,28,29].

### 6.2. Impacts of These Mechanisms and Their Relationship to Innate and Adaptive Immune Systems

The regulatory mechanisms of the immune system, especially those involving regulatory T cells (Tregs), are crucial for preserving immunological homeostasis and averting overreactions or detrimental immune reactions. Tregs lower the likelihood of chronic inflammation and autoimmune responses by suppressing immune cell overactivation. By inhibiting effector T cells (adaptive) and modifying antigen-presenting cells (innate), they serve as a link between innate and adaptive immunity, guaranteeing balanced reactions.

These systems are essential for immune defense cooperation. A specialized, long-lasting defense is tailored by adaptive immunity, whereas innate immunity starts responses and exposes adaptive cells to antigens. My emphasis on molecular immune regulation is closely related to the regulatory pathways that guarantee the regulation of this interaction, avoiding collateral harm and preserving tolerance to self-antigens. These pathways include cytokine signaling and Tregs.

## 7. Function and Dysfunction of Regulatory T Cells

Regulatory T cells (Tregs) are mainly characterized by representations of CD25, CD4, and FoxP3 transcription factors. Tregs play an important role in preventing autoimmunity and establishing a balance in immune homeostasis. Specifically, the regulatory T cells restrict immunological responses by preventing effector T cells, dendritic cells, and other immune cells from proliferating and producing cytokines. The representation of immune checkpoint molecules such as CTLA-4, production of regulatory cytokines like TGF-β and IL-10, and regulation of metabolic pathways via molecules such as CD73 and CD39 are the various techniques in which suppression occurs [30,31,32,33]. Furthermore, during inflammation and injury, Tregs preserve tissue homeostasis, guaranteeing a well-balanced immune response that lessens collateral damage. In specialized tissues like the skin and gut, where the immune system is continuously interacting with external antigens, they are especially important.

Treg dysfunction manifests in a vast spectrum of illnesses. Exaggerated immune responses caused by deficiencies in Treg function or numbers frequently result in autoimmune illnesses such as multiple sclerosis, systemic lupus erythematosus, and type 1 diabetes. FoxP3 gene mutation processes can result in acute immunopathologies, such as polyendocrinopathy, dysregulation of immunity, X-linked syndrome (IPEX), and enteropathy [34,35]. On the other hand, an overactive Treg population can severely inhibit immune responses, which can worsen chronic infections and aid in cancer tumor immune evasion. For example, cytotoxic T cells can be inhibited by Tregs in the tumor microenvironment, allowing the tumor to grow. Tregs’ ability to balance and change between pro- and anti-inflammatory states is essential to their function. To restore immunological balance in disease situations, treatment techniques that target Tregs must be developed with an understanding of these dynamics.

### Interactions Between Regulatory T Cells

The interplay between suppressor T cells encompasses both microenvironment modulation and direct cellular interactions. Effector T cells communicate with Tregs through cell-to-cell interaction controlled by molecules such as CTLA-4, which reduces the regulation of costimulatory signals that present antigens [16]. Additionally, they generate cytokines that reduce inflammation and immune cell recruitment, like IL-10. By upsetting the stability of other Tregs, dysregulated Tregs cause FoxP3 expression to decline and a change toward an effector phenotype. The immunological landscape is shaped by this intricate interaction, which affects both health and disease outcomes.

## 8. Materials and Methods

**Figure 1:**
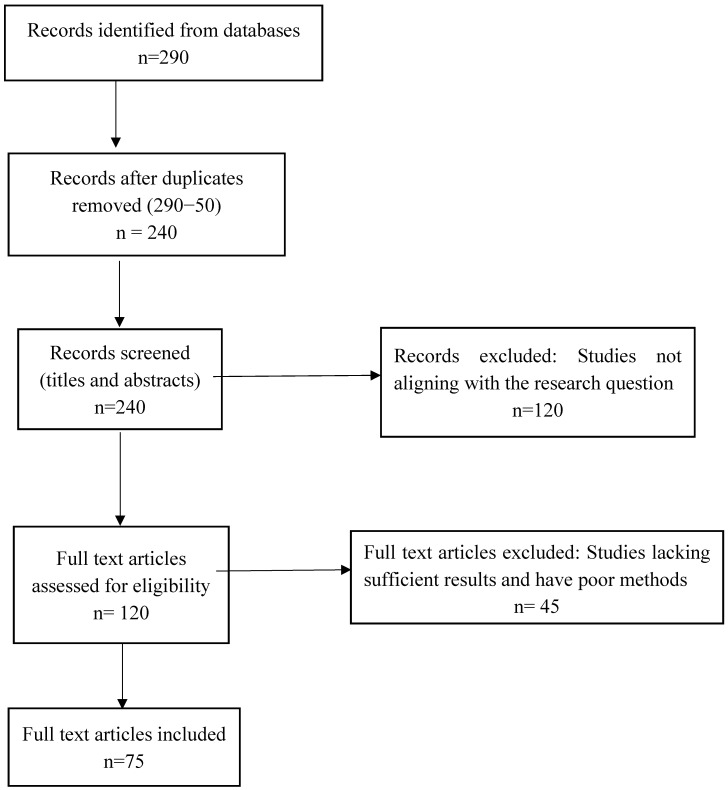
PRISMA flow diagram.

A thorough search of the literature was done to find publications in the PubMed database. Molecular mechanisms, immunological regulation, immune signaling pathways, cytokine signaling, and immune homeostasis were among the keywords used to find relevant articles. With an emphasis on those released within the last three years, publications from 2014–2024 were included. In particular, the inclusion criteria called for studies that provide fresh proof of immunity-related molecular regulation. As a result, 75 papers were chosen for this review. Figure 1 provides an overview of the literature selection procedure.

### 8.1. Study Design and Methodology

Using a systematic review methodology based on PRISMA criteria, this paper examines recent developments in molecular immune modulation. Database searches were used to find pertinent studies, which were then vetted by full-text reviews and filtered by title and abstract. Studies that looked into immune regulation mechanisms, namely immunological homeostasis, cytokine signaling, or immune pathways, and were published between 2014 and 2024, with an emphasis on the last three years, were included. Peer-reviewed papers from respectable journals were the only ones taken into account. Duplicate records, inadequate molecular data, and non-mammalian studies unless pertinent were among the exclusion criteria. The results advance our knowledge of immune system control by offering fresh perspectives on important immunological mechanisms.

### 8.2. Data Collection and Analysis

To reduce bias, data extraction was carried out separately by two reviewers. The study’s goals, methods, main conclusions, and important findings were all methodically gathered and organized in a structured spreadsheet. Any disagreements among the reviewers were settled by dialogue or by consulting a third party. Trends in immune regulation research were assessed using a qualitative method for data synthesis. Based on their main areas of interest, such as immunological homeostasis, cytokine signaling pathways, and molecular immune interactions, the studies were grouped. Analysis of the results revealed new ideas, parallels, and inconsistencies in the literature.

## 9. Review Themes

The six themes discussed in this review include T-cell and Checkpoint Regulation, Epigenetic Regulation, Cytokine Signaling Pathways, Suppressor T Cells, Immunometabolism, and Regulatory T Cells. In a review of the molecular mechanism of immune regulation, there is a connection between these themes. Cytokine signaling pathways are considered the main communication avenue for the immune system, interfacing responses between adaptive and innate immunity. Cytokines such as TGF-β and IL-2 are crucial for the function and differentiation of suppressive T cells and Tregs, preventing inflammation and improving immune tolerance. Besides, cytokines impact the representation of checkpoint molecules like CTLA-4 and PD-1, which regulate the activity of T-cells and establish a balance in the immune system [36,37,38,39]. This is a crucial interaction underscoring the importance of signaling pathways in improving cellular immune responses.

There is a close relationship between the functions of suppressor T cells and regulatory T cells (Tregs) concerning the modulation of immune activity. Regulatory cells suppress responses of effector T cells and foster tolerance through the production of cell-to-cell contact or cytokines [40]. On the other hand, suppressor T cells employ distinct techniques, like disruption of metabolism or direct cytolysis, to downgrade activation of immune function. Histone acetylation and DNA methylation are two examples of the complex epigenetic changes that control both cell types and guarantee their stability and functionality in a variety of physiological and pathological circumstances. For instance, FoxP3, a master regulator of Tregs, is tightly regulated by epigenetics, which connects immunological function and cellular growth. Therefore, a common framework for these subjects is provided by the interaction of various biological systems and how they are regulated.

By illustrating how metabolic changes impact immune control, immunometabolism connects these processes. Regulatory T cells depend on oxidative phosphorylation when executing suppressive tasks. On the other hand, effector cells rely on glycolysis for faster proliferation. Gene expression and immunological function are linked to cellular energy levels by epigenetic regulators, which frequently react to metabolic stimuli [41,42,43,44]. These themes come together to show a delicate yet well-organized web of signaling, control, and metabolic adaptation that is essential for preserving immunological homeostasis and treating dysfunction in illnesses like cancer and autoimmune diseases. This interwoven story highlights the common structure that underlies these disparate ideas.

## 10. Results

### 10.1. Cytokine Signaling Pathways

Among the critical factors implicated in immune system regulation, cytokines are key, particularly in mediating information flow from one immune cell to another. However, not all cytokines participate in such regulation. The specific cytokines that have been identified as key include interleukin (IL)-10, IL-17, and IL-23. In particular, IL-10 plays an important role in immune system regulation: this anti-inflammatory cytokine prevents damage to vital tissues by limiting inflammatory responses. Notably, IL-10 directly suppresses macrophage activation while inhibiting pro-inflammatory cytokine production [12,13].

In contrast, IL-17 plays a unique role in stimulating inflammatory responses in autoimmune diseases such as psoriasis and arthritis [14,15]. IL-17 is responsible for promoting neutrophil infiltration and activating the inflammatory processes observed in autoimmune diseases [16,18]. Thus, the competition between pro-inflammatory and anti-inflammatory cytokines is pivotal to immunity.

IL-23, another vital cytokine involved in immune regulation, has been implicated in T helper 17 (Th17) generation [19] and proliferation [20]. Th17 cells are involved in inflammatory disorders such as Crohn’s disease and multiple sclerosis; thus, targeting IL-23 has emerged as a therapeutic approach for these conditions [21,22]. Exploring the mechanisms by which these cytokines are regulated will offer insights into how they contribute to maintaining a healthy immune system and increase our understanding of disease processes.

The discussion of diversified functions of the cytokine interleukin-6 (IL-6) focuses on its multiple anti-inflammatory and pro-inflammatory functions in the roles of the immune system. The results of this study underscore the crucial function of interleukin-6 (IL-6) as an element with diversified function in the modulation of the immune response, especially in the pathogenesis of autoimmune diseases and inflammation [45,46,47,48]. High levels of IL-6 were associated with autoimmune and inflammatory diseases, underscoring its centrality in the progression of these diseases. In addition, the study highlighted the therapeutic abilities of IL-6 inhibitors, including siltuximab and tocilizumab, in addressing autoimmune diseases, cytokine release syndrome, and hyperinflammation induced by COVID-19. Moreover, pathways that target IL-6 signaling, such as JAK or STAT, showed effectiveness in mitigating inflammatory responses. These results demonstrate the significance of establishing a balance in IL-6 regulation to reduce severe effects while capitalizing on its therapeutic promise in various immune-mediated conditions.

It is crucial to target specific pathways and immune cells to foster cancer immunotherapy. It has been demonstrated that Th2 cells, which are controlled by cytokines such as IL-25 and IL-33, stimulate tumorigenesis and metastasis, and that pharmacological inhibition successfully prevents tumor growth. Th17 cells have been shown to decrease anti-tumor immunity in colorectal cancer (CRC), indicating context-dependent targeting techniques, despite their favorable effects in certain malignancies, such as melanoma [49]. In preclinical settings, activating B cells with substances like CD40 stimulation and CpG-ODN demonstrated promise in improving T-cell responses and lowering tumor burden. On the other hand, Breg cells are involved in immunosuppressive tumor microenvironments (TMEs), and preclinical research showed promise in inhibiting them using substances like resveratrol and LXA4. Together, these results demonstrate the dual function of inflammation in cancer and support the use of specialized strategies to alter immune responses to enhance treatment results. In a variety of physiological and pathological settings, cytokine signaling pathways play a crucial role as immunological communication mediators, coordinating the equilibrium between inflammation and tolerance [50].

### 10.2. T-Cell and Checkpoint Regulation

Adaptive immunity is a complex mechanism that involves interplay among various components, including T cells, that are critical for maintaining homeostasis through regulatory processes. Recent research has focused on adaptive immunity, emphasizing immune checkpoints such as programmed cell death ligand (PD-1/PD-L1) and cytotoxic T-lymphocyte-associated protein 4 (CTLA-4). These factors are critical because they prevent autoimmunity and help achieve and maintain immune tolerance [23,24,25]. The PD-1 receptor expressed on T cells interacts with PD-L1 and PD-L2 on antigen-presenting cells (APCs) to hinder T-cell activation [26,27,28]. The PD-1/PD-L1 axis is essential for maintaining tolerance and thus safeguarding against tissue harm during inflammation.

CTLA-4 is another checkpoint molecule that hinders T-cell activation by competing with CD28 for binding to B7 ligands on APCs [29,30,31]. This competition leads to decreases in stimulatory signaling and T-cell proliferation.

Research has shown that additional immune checkpoint proteins—T-cell immunoglobulin, mucin-domain containing-3 (TIM-3) and Lymphocyte Activation Gene 3 (LAG-3)—regulate T-cell activity. TIM-3 has been shown to suppress the Th1 cell-specific response, resulting in the production of regulatory T cells (Tregs) [32,33,34]. LAG-3 suppresses T-cell activation through interactions with MHC class II molecules to restrict T-cell activation while decreasing cytokine synthesis [35,36]. Studying these immune checkpoints will contribute to understanding immunity and produce potential therapeutic approaches in oncology and autoimmunity.

Oncogenic pathways, inflammatory signaling, and epigenetic mechanisms influence the regulation of CTLA-4 expression and PD-L1 at the Ribonucleic Acid (RNA) level, fostering a version of immunity in cancer [47]. The emergence of FN-γ is a vital inducer of PD-L1 through the pathways of JAK-STAT-IRF1. However, other cytokines such as IL-17 and IL-6 foster the expression of PD-L1 via various signaling cascades. Additionally, PD-L1 transcription is dynamically modulated by oncogenic pathways such as MAPK, EGRF, and PI3K-AKT, fostering suppression of immunity. Moreover, miRNA and m6A methylation are among epigenetic regulators that post-transcriptionally impact the expression of PD-L1. In general, these mechanisms underscore the intricacy of the regulation of immune checkpoints, providing crucial therapeutic targets to foster the effectiveness of immunotherapy [47].

CD8+ T-cells perform a crucial role in protecting the body against tumors and improving the role of immune checkpoint inhibitors (ICIs) [48]. The process of activating T-cells involves various important steps; determination of the type of antigen, migration, and effector stages. The components of immune checkpoints such as PD-1 and CTLA-4 modulate the T-cells activation process [38]. ICI resistance is associated with disruption of migration of T-cells, antigen presentation, and functions of effectors. Besides, suppressive immune cells, compromised chemokine signals, and reduced expression of MHC foster resistance. Combination therapeutic techniques targeting these processes are under research. Therefore, the complex interaction between immune checkpoint pathways and T-cell activation highlights the importance of these pathways in regulating immunological responses and offers a possible treatment option for immune-related illnesses [50].

### 10.3. Mechanisms of Immune Regulation

#### 10.3.1. Regulatory T Cells: Mechanisms of Regulation

Tregs play an important role in immune tolerance by suppressing other immune cells to prevent autoimmunity [3]. Cytokines such as IL-2 are critical for the development, proliferation, and regulation of Tregs. Tregs are characterized by the expression of FoxP3, a transcription factor involved in cell development and function [3]. Treg stability is also controlled by metabolic processes, such as fatty acid oxidation (FAO) under low-fat conditions [7]. Signals derived from tumor necrosis factor receptor (TNFR) and inhibitory receptors, including CTLA-4, stringently modulate Treg function and prevent the overactivation that can cause tissue-damaging inflammation [29,30,31].

PRMT5 and its similar pathways play a crucial role in immunosuppression and Treg cell function. In essence, PRMT5, via its control reciprocal of the recruitment of STAT3/STAT5 and FOXP3, is important in influencing the differentiation of Th17 and the maintenance of Treg activity. Conditional deletion of PRMT5 plays a critical role in the disruption of Treg cells’ suppressive functions, causing phenotypes that resemble autoimmune factors. In addition, in breast cancer models, PRMT5 inhibitors foster anti-tumor immunity [51,52,53]. Moreover, Treg cells induce suppression via various techniques, such as extracellular vesicle (EV) communication, metabolic modulation, production of cytolysis (including IL-10, TGF-β, and IL-35), and perforin/granzyme-mediated cytolysis. These results underscore the therapeutic potentials of PRMT5 in the modulation of Treg functions during cancer and autoimmune settings.

Various regulatory mechanisms and functional subsets of T cells play a crucial role in boosting Treg immune function and tolerance. In particular, Th9 cells, with the influence of IRF4 and PU.1, remain undiscussed as a distinct lineage. Trl cells, defined by the production of IL-10, depend on factors like c-Maf and Blimp 1 but do not have firm lineage markers. Tregs possess Th-like characteristics under inflammation conditions, including RORγt and T-bet expressions, affecting their suppression and migration. Innate lymphoid cells and γδ T cells play important functions in primary immune responses; ILCs resemble Th subsets and, like γδ T cells, release IL-17. These results highlight the complex balance of tolerance, immunity, and the requirement for further studies to evaluate therapeutic targets. With their many functions in preserving tolerance and averting autoimmunity, regulatory T cells continue to be at the forefront of immunological regulation, offering a fundamental basis for comprehending and regulating immune homeostasis [54,55,56].

#### 10.3.2. Suppressor T Cells: Mechanisms of Regulation

Suppressor T cells include subsets such as Tr1 cells that suppress immune responses through contact with cytokine-producing cells and are thus critical for immune regulation [3]. Unlike Tregs, suppressor T cells are not dependent on FoxP3 but often produce the immunosuppressive cytokines IL-10 and TGF-β [3]. The regulation of suppressor T-cell number and activity depends on the microenvironment, such as the presence of inflammatory cytokines and metabolic factors. Suppressor T cells are modulated through surface molecules such as PD-1 and LAG-3, which help maintain their suppressive function in inflamed tissues and prevent overactive immunity [35,36].

Moreover, regulatory T-CELLS (Treg cells) perform a critical role in immune regulation because of their dynamic effector and trafficking mechanisms. The study determined that chemokine receptors such as GPR15, CCR6, and CXCR4 are crucial in guiding T-cells to barrier tissues such as intestines, skin, and bone marrow; these receptors control the immune response and foster tolerance [54]. Specific subsets, including follicular T-cells, modulate T and B helper cells through specialized techniques. The ability of Treg cells to sustain tolerance of peripheral immunity is associated with their timely thymic egress, pre-activated state, and environmental adaptations. These results foster the understanding of the crucial role of Treg cells in immune response.

Treg cells perform a crucial role in tissue repair, immune regulation, and disease progression in various contexts, including autoimmunity, cancer, pregnancy, aging, and chronic infection [57]. Overactivity or dysfunction of Treg cells is associated with the progression of cancer, autoimmune diseases, and impaired efficacy of vaccines. Treg accumulates in aging, but depicts compromised functionality, resulting in inflame-aging, dysregulation of immunity, vulnerability to infections, tumor growth, and development of autoimmune diseases. The study highlights the intricacy of Treg, environmental, and tissue-specific conditions, stressing their multiple functions as critical contributors to pathological processes and protectors of immune homeostasis. The knowledge of the diversity of Treg cells provides crucial therapeutic techniques [58].

Suppressor T cells (Tregs) employ various techniques to regulate antitumor immune response. Tregs hinder effector T cell activation by interfering with costimulatory signaling through the use of cytotoxic T lymphocyte antigen 4 (CTLA-4) [48]. They can dominate IL-2 consumption thanks to their high-affinity receptor for IL-2, which promotes their accumulation in the tumor microenvironment (TME) at the expense of effector T cells. Tregs further reduce antitumor immunity by secreting immunosuppressive cytokines such as TGF-β, IL-10, and IL-35 [51]. Additionally, Tregs take advantage of metabolic changes that enable them to flourish in the nutrient-poor TME, including oxidative phosphorylation and fatty acid consumption. These complex processes make immunotherapies like PD-1 blockage less effective, underscoring the possibility of using Treg targeting as a tactic to improve cancer therapy. By preserving immunological balance and showing therapeutic promise in the management of immune-mediated illnesses, suppressor T cells provide an excellent example of the intricacy of immune regulation [59].

#### 10.3.3. Epigenetic Regulation

It is becoming apparent that epigenetic changes are critical modulators of immunity. Epigenetic mechanisms, such as DNA methylation, histone modifications, and non-coding RNAs (ncRNAs), impact gene expression in immune cells [37,38,39]. DNA methylation can either suppress or increase immune-related gene expression, depending on the context [40]. Irregular DNA methylation patterns are linked to conditions such as systemic lupus erythematosus (SLE) and rheumatoid arthritis [41,42]. Histone modifications, such as acetylation and methylation, also impact gene expression. Histone acetylation is linked to open chromatin and an active transcription state, whereas methylation can result in either activation or repression of gene expression based on the particular histone residue. Enzymes that modify histones at the level of gene expression play a role in modulating the immune response.

Additional mechanisms of epigenetic regulation have also been reported in immune cells. Epitranscriptomics has revealed that N6-methyladenosine (m6A) is involved in the stability and translation of immune-related mRNAs. Proteins within chromatin remodeling complexes, including switch/sucrose nonfermenting (SWI/SNF), regulate T-cell differentiation and macrophage activation by controlling access to immune-related genes. Knowledge of these epigenetic regulators highlights the complex regulation of gene expression related to the immune system, and the data offer new insights into the dysregulation of immune responses in disease.

MicroRNAs (miRNAs) constitute a long-term defense system. For example, miRNAs such as miR-146A and miR-155 actively influence the immune response by targeting key signaling molecules. Research has demonstrated that miR-146A can inhibit NF-κB signaling by targeting TRAF6 and IRAKI to dampen the production of inflammatory cytokines [5,43]. Similarly, miR-155 contributes to inflammation by targeting the suppressor of cytokine signaling (SOCS), which leads to an increase in inflammatory cytokine production.

The other miRNAs involved in immune regulation include miR-21, which controls T-cell activation through PTEN and whose expression is regulated by Tim-3; miR-223, which regulates macrophage polarization in inflammation; miR-181b, which plays a role in adjusting T-cell sensitivity; and miR-125b, which regulates cytokines by influencing pro-inflammatory signaling molecules. These miRNAs, by functioning as key regulators of immune and inflammatory processes, represent how ncRNAs impact the immune response.

lncRNAs play a role in controlling the immune system, as evidenced by the impact of the lncRNA NeST on interferon-gamma levels through interactions with chromatin modifiers to shape the development of Th1 cells [44]. The dysregulation of ncRNAs can lead to autoimmune and inflammatory issues, indicating the necessary role of such factors in the normal function of the immune system. Although some advancements have been made, many aspects of epigenetic regulation, including how ncRNAs, chromatin remodelers, and RNA modifications interact, remain poorly understood.

In immune evasion, progression of tumors, and therapy resistance, epigenetics plays a critical role. Tumor-infiltrating Treg cells have significantly high levels of H3K27 and EZH2, triggering immunosuppressive environments, and CCR6-CCL20 pathways produce Th17 cells that complicate the prognosis [58]. The gut microbiome affects cancer immunotherapy and Treg cell recruitment, especially through compounds like butyrate. Through epigenetic processes, cancer stem cells (CSCs) enhance tumor recurrence and metastasis by promoting intratumoral heterogeneity (ITH) and immune resistance. Although there are still issues, clinical trials show that epigenetic medications that target DNMT, EZH2, and HDAC have anticancer potential [50]. One promising approach to cancer treatment is the combination of immunotherapy and epigenetic modification [60].

MicroRNAs (miRNAs) and long non-coding RNAs (lncRNAs) play a key role in regulating liver disease, especially non-alcoholic steatohepatitis (NASH) and hepatocellular carcinoma (HCC). While lncRNA-ROR encourages HCC metastasis, lncRNAs such as lnc-DILC inhibit the growth of liver cancer stem cells. Although the processes are still unknown, lncRNAs such as lnc18q22.2 affect hepatocyte survival and the oxidative stress response in NASH. In Kupffer cells (KCs), miRNAs regulate inflammation [59]. For example, miR-155 decreases NFκB-driven signaling, while miR-192-5p increases inflammation by intercellular communication. LXR agonists, vitamin D receptor activators, and Hippo pathway modulators are examples of therapeutic targets that have shown promise in reducing the inflammation, fibrosis, and metabolic dysregulation linked to NASH. Therefore, a dynamic layer of immune control is provided by epigenetic alterations, which emphasize the possibility of modifying gene expression to alter immune responses in conditions including cancer, autoimmune illness, and others.

The functions of several microRNAs (miRNAs) in immune regulation, together with their targets, impacted cell types, and pathways, are displayed in Table 1. While miR-155 promotes the production of cytokines, miR-146a inhibits inflammation through NF-κB. While miR-223 and miR-222 control macrophage polarization, miR-21 and miR-10a encourage the formation of Tregs and immunological tolerance. miR-150 regulates B/T cell development, while miR-181 increases T-cell receptor sensitivity. Together, these miRNAs affect immunological responses, inflammation, and tolerance, which in turn affects immune-mediated diseases, disease progression, and immune homeostasis.

#### 10.3.4. Immunometabolism

Immunometabolism is the process whereby immune cells undergo metabolic changes—activation, differentiation, and function—to fulfill necessary biological roles. Signaling pathways that affect metabolic processes, such as glycolysis, oxidative phosphorylation (OXPHOS), and fatty acid metabolism, are intricately associated with fluctuations in the immune response. These metabolic processes and pathways create energy-dependent and inflammatory environments for immune cells.

Immunometabolism plays an important role in fostering immune functions by bringing immunity and metabolism, emphasizing how metabolic processes improve systemic health and immune cell function [60]. The bidirectional connection between metabolism and immunological responses is one of the main discoveries; metabolic reprogramming affects tissue homeostasis, macrophage activity, and T and B cell activation. Immune states are influenced by environmental signals like nutrition and metabolic processes including lipid metabolism and glycolysis in situations like obesity, cancer, and inflammation. Complex networks within and between cells have been revealed by advances in computational and experimental methods. Potential treatment approaches for cancer, metabolic disorders, and immune-mediated diseases are presented by this knowledge [60].

Microphage metabolism is greatly influenced by viral infections, thus the need for various pathways including lipid metabolism, glycolysis, and amino acid. Viral replication is associated with increased glycolysis in HIV, Dengue, and SARS-CoV-2 infections, with different levels of glycolytic intermediates depending on the stage of infection and macrophage state. Viral survival and immune responses are supported by lipid metabolism, which is regulated by signaling pathways such as MyD88 and TRIF [70]. Cholesterol is essential for viral entry and inflammation, particularly during SARS-CoV-2 infection [61]. Amino acid metabolism, specifically glutamine and arginine, supports viral replication and immune modulation. These findings point to metabolic reprogramming as a possible therapeutic target in viral infections.

Homeostatic regulation is an important process in immunometabolism because it helps establish a balance in immune cell functionality. mTORC1 is a key regulator that controls autophagy and anabolic activities according to nutrition availability [62]. Lipid and glucose metabolism is influenced by SREBPs and LKB1, which impacts T cell differentiation and proliferation. Immune cell metabolism is disturbed by the tumor microenvironment; T cell activity is hampered by glucose deprivation, but fatty acid intake meets energy requirements. Through tryptophan depletion, NAD metabolism, and immunosuppressive metabolites, the CD38 and IDO pathways further control immunological responses, affecting T cell function and promoting immune tolerance. These processes highlight the intricate relationship between immunity and metabolism. Therefore, the new discipline of immunometabolism provides novel insights into immune regulation and possible targets for therapeutic approaches by exposing the significant influence of metabolic reprogramming on immune cell function.

### 10.4. Cellular Interaction

#### 10.4.1. Effector T Cells

Effector T cells predominantly utilize glycolysis for energy generation, biosynthesis upon activation, and cytokine secretion. Potential functional glycolytic genes include HK2, PFKFB3, and MYC, which regulate glycolytic flux. This flux enables rapid adenosine triphosphate (ATP) generation, even under the hypoxic conditions often found in inflamed tissues, helping fulfill the high energy requirements of proliferative and functional effector T cells [63,64].

#### 10.4.2. Tregs

Tregs depend on the OXPHOS and FAO pathways for their suppressive functions. Peroxisome proliferator-activated receptor gamma coactivator 1-alpha (PPARGC1A) and FOXP3 are crucial for the metabolic preference of Tregs for OXPHOS and FAO [63,65]. FAO permits Tregs to function under the low-glucose conditions present in chronic inflammation [64,66].

#### 10.4.3. Macrophage Polarization

Polarized macrophages differ in their metabolic behavior depending on their state. M1 macrophages rely on glycolysis for their pro-inflammatory functions, and the genes required for these functions are activated by hypoxia-inducible factor 1 alpha (HIF1A) and lactate dehydrogenase A (LDHA). Conversely, M2 macrophages support OXPHOS and FAO through the adipogenic factor peroxisome proliferator-activated receptor gamma (PPARγ) and the carcinogenic factor ATP citrate lyase (ACLY) to execute restorative and anti-inflammatory effects [65,66].

#### 10.4.4. Key Signaling Pathways

The mechanistic targets of rapamycin (mTOR) and AMP-activated protein kinase (AMPK) pathways, a cellular energy sensor responsible for the maintenance of energy homeostasis, represent important regulators of immunometabolism. mTOR, encoded by MTOR, promotes glycolytic pathways and effector T-cell function. Conversely, AMPK, encoded by PRKAA1, is involved in various catabolic processes, such as FAO, and maintains Treg stability [63,64]. Dysregulation of the mTOR and AMPK pathways leads to immune imbalance in various autoimmune diseases and metabolic disorders [65,66].

Key immune regulatory mechanisms, their corresponding genes, regulators, impacted cell types, and functions are displayed in Table 2. IL6, IL10, and TGFB1 are involved in cytokine signaling, which uses pro- and anti-inflammatory cytokines to balance inflammation. FOXP3 and IL2RA control regulatory T cells (Tregs), which reduce immunological responses and increase tolerance. Histone acetylation and DNA methylation are two mechanisms by which epigenetic changes, such as HDAC9 and DNMT3A, affect T-cell and macrophage development. Together, these systems control immune responses, preserving immunological homeostasis and avoiding dysregulation [58,59].

Immune regulatory mechanisms, their flaws, related illnesses, and pathophysiological effects are presented in Table 3. By encouraging inflammation, cytokine signaling abnormalities, like increased IL-6, are linked to rheumatoid arthritis and cytokine storms. Autoimmunity results from FOXP3 mutations that affect Treg function. Autoimmune tolerance is upset by epigenetic changes and mTOR hyperactivity, leading to metabolic and autoimmune illnesses. Because impaired autophagy decreases pathogen clearance, it plays a role in neurological illnesses and Crohn’s disease. These processes show how a variety of disease disorders are caused by immunological dysregulation [60,61,62].

## 11. Discussion

Recently, there has been an increasing focus on deciphering the regulatory functions of ncRNAs in immune regulation. In vitro studies have revealed that miRNAs regulate cytokine levels and immune cell differentiation [67,68]. For example, there is evidence indicating that miR-146a is involved in regulating the responses to inflammation by targeting the NF-κB signaling pathway [69]. Additionally, similar to miRNAs, lncRNAs can regulate immune responses by interacting with chromatin modifiers and modulating the expression of immune-related genes [70,71]. Epigenetic regulation has been identified as a major determinant of immune balance [72]. Future research is needed to clarify how these mechanisms connect and contribute to immune homeostasis under normal and pathological conditions. New immune regulators, including DNA methyltransferases and histone-modifying enzymes, have also been identified [73]. These regulators influence the accessibility of immune-related genes that direct immune cell differentiation and functionality. For example, DNA methylation is involved in controlling the expression of genes encoding cytokines, and its dysregulation is implicated in diseases such as SLE and rheumatoid arthritis [74].

### 11.1. Control of Immune Responses by MicroRNA

The influence of microRNAs (miRNAs) on the regulation of immune response is manifested in their impact on crucial immune cell functions and signaling pathways (Table 1). For instance, in inflammatory responses, miR-146a and miR-155 have opposite effects, with miR-146a suppressing inflammation and miR-155 boosting it. These results emphasize how crucial post-transcriptional regulation is for maintaining a balance between tolerance and immune activation. Furthermore, miR-223 and miR-181 have an impact on T-cell receptor sensitivity and macrophage polarization, respectively, indicating their role in adaptive immunity. Therapeutic approaches aimed at dysregulated immune responses in autoimmune and inflammatory illnesses can be improved by an understanding of these molecular regulators.

### 11.2. Molecular Processes Controlling the Immune System

Table 2 lists a number of interrelated biological pathways, such as cytokine signaling, immunological checkpoints, and epigenetic alterations, that control immune regulation. Immune homeostasis is directly impacted by cytokine-mediated signaling, including IL-6 and TGF-β, which either stimulate or inhibit inflammation. While suppressor T cells use IL-10 and TGF-β signaling to promote immunological tolerance, regulatory T cells (Tregs), which are identified by their expression of FOXP3, are essential in preventing autoimmunity. Furthermore, immune gene expression is modulated by epigenetic mechanisms such as DNA methylation and histone acetylation, underscoring the complex regulation of immunological responses. These results imply that immune regulation is a multifaceted, intricate process that can be modulated therapeutically.

### 11.3. Immune Mechanism Dysregulation in Disease

Diseases linked to abnormalities in cytokine signaling, immunological checkpoints, and metabolic pathways clearly exhibit the pathogenic effects of immune dysregulation (Table 3). The negative consequences of excessive inflammation are highlighted by the fact that cytokine storm syndromes and rheumatoid arthritis are characterized by an overproduction of IL-6. Similarly, cancer and autoimmune illnesses are influenced by immune checkpoint malfunction, specifically with regard to PD-1 and CTLA-4. Immune abnormalities, as observed in multiple sclerosis and lupus, are further aggravated by dysregulated miRNA expression and epigenetic changes. Metabolic disorders are also linked to abnormal immunometabolism, including mTOR hyperactivity. These results highlight the need for tailored treatments that restore immunological balance in illness settings.

### 11.4. Immunometabolism and Immunotolerance

Several studies have described the phenomenon by which metabolic reprogramming controls immune functions [75,76]. In general, metabolic pathways such as glycolysis and FAO contribute to the differentiation and activation of T cells and macrophages [77]. In addition to playing an active role in cell fate, these metabolic pathways support various cellular processes by supplying the energy and biochemicals essential for cell functions. For example, effector T cells use glycolysis instead of OXPHOS since considerable energy is required for proliferation and cytokine production [66,67,68,69,70,71,72]. This metabolic reprogramming allows effector T cells to function under the hypoxic and high-energy-demand conditions common to inflamed tissues. In contrast, Tregs require fatty acids and OXPHOS for their suppressive functions. Therefore, Tregs remain stable even under low-glucose concentrations, as is common in chronic inflammatory diseases. The metabolic plasticity of the T cell is a fundamental that allows a particular immune response to the inflammatory situation and microenvironment. This interaction between immunometabolic pathways and other regulatory elements, such as epigenetic modifications and immune checkpoints, remains one of the most important areas for further research. It has to be underlined that in several diseases, many of the mechanisms described here may coexist and contribute to the disease pathogenesis. This cumulative effect highlights the complexity of disease etiology and the interplay between molecular pathways.

Studies on metabolism and the immune system have provided new potential approaches for disease management. Modifying immune responses by targeting metabolic pathways, such as the mTOR and AMPK pathways, is useful in conditions governed by autoimmunity and inflammation. mTOR, for example, controls T-cell activation, differentiation, and metabolic programming contingent on nutrient status. In autoimmune diseases, immune cell activity is often excessive; therefore, mTOR inhibition can potentially limit effector T-cell-associated inflammation [75]. In contrast, the activation of AMPK simultaneously favors catabolic pathways, Treg stability, and pro-inflammatory activity [78]. Therefore, it may be possible to therapeutically influence these metabolic pathways in autoimmune inflammatory diseases. Future studies should further elucidate the exact mechanisms by which immunometabolic signaling is modulated to refine treatment strategies in disease settings in which metabolic dysregulation plays a major role.

### 11.5. Implications for Therapy and Illness

Numerous illnesses, including infections, cancer, autoimmune disorders, and chronic inflammation, are exacerbated by the dysregulation of immunological regulatory systems. In diseases like rheumatoid arthritis and inflammatory bowel disease, excessive inflammation is caused by improper inflammasome activation, whereas aberrant autophagy affects antigen presentation and results in autoimmunity. Mitochondrial failure promotes metabolic disorders and immunological fatigue by changing the metabolism of immune cells.

By regulating inflammatory responses and immunological tolerance in aging and neurodegenerative disorders, sirtuin targeting has therapeutic potential. Post-translational changes impact the results of cancer immunotherapy by controlling cytokine signaling and immunological checkpoints. In autoimmune and infectious disorders, microRNAs function as biomarkers and therapeutic targets for immune regulation [59].

The goal of precision medicine techniques such as gene-editing technology, biologics, and small-molecule inhibitors is to reestablish immunological homeostasis. Cancer treatment is being revolutionized by personalized immunotherapies including adoptive cell therapy and checkpoint inhibitors. Comprehending these pathways facilitates the development of innovative therapeutic approaches for the efficient treatment of immune-related illnesses.

## 12. Review Question

Research on immunological modulation should continue to be shaped in the future by combining historical viewpoints with current discoveries. Addressing unresolved issues and opening the door for novel therapeutic approaches against immune-mediated illnesses require such an integrative strategy. In this area, one important unanswered question is how exactly regulatory T cells (Tregs) preserve immunological homeostasis without pathologically suppressing protective immune responses. Tregs are well-known for their ability to prevent autoimmunity, but when they malfunction or become overactive, they can cause autoimmune illnesses or compromise anti-tumor immunity, creating a delicate balance that is difficult to achieve [58].

The interaction between regulatory Tregs and other regulatory cells, like epigenetic modification and cytokines, is not well-understood. Addressing this intricacy needs a precise evaluation of the effects of metabolic and environmental factors on Treg stability and functionality. For instance, understanding the chemical signals that dictate whether Tregs inhibit or allow immunological responses may provide fresh perspectives on how to modify these cells for therapeutic ends. Molecular, epigenetic and immunometabolic viewpoints might be combined in an integrated manner to show how these pathways interact to affect Treg behavior. To fill in existing knowledge gaps and meet new clinical demands, these discoveries have the potential to greatly influence the creation of focused strategies for modifying Tregs in the context of cancer, autoimmunity, and transplantation.

### 12.1. Limitations of the Study

This study has several drawbacks that should be recognized. First, bias may be introduced by differences in study design, sample size, and methodology among various sources, even if a large body of the literature was examined. Furthermore, the majority of research focuses on certain immune regulation pathways, which prevents a thorough integration of all molecular mechanisms. Direct experimental validation of results is also limited by the dependence on secondary data. Furthermore, because of publication lag, new findings in immune regulation may not be fully represented, especially in fields like immunometabolism and non-coding RNAs. For a more comprehensive understanding, future research should use a standardized methodology and more recent data.

### 12.2. Future of the Study

Future studies should integrate multi-omics techniques, such as transcriptomics, proteomics, and metabolomics, to deepen our understanding of molecular immune modulation. Examining how immunological checkpoints, regulatory T cells, and epigenetic changes interact to cause disease progression may help identify new targets for treatment. Furthermore, investigating the ways in which environmental factors impact immune regulation could improve personalized medical strategies. Translating research into successful treatments will require clinical trials assessing immunomodulatory medicines that target important biological pathways. Last but not least, developments in machine learning and artificial intelligence may improve predictive modeling, making it easier to spot immune dysregulation trends in a range of illnesses.

## 13. Conclusions

The healthcare industry has learned new things about cellular-level molecular immunomodulatory mechanisms within the past decade. Epigenetic regulation, immunometabolism, cytokines, and immune checkpoints are some of the fundamental immunological components that preserve immunological homeostasis. Given their dynamic connections, these elements point to a fresh perspective on how immune systems react to shifts in homeostasis and disease conditions. Therefore, developing novel treatment strategies that alter these molecular targets may be beneficial in the management of inflammatory and autoimmune processes as well as the diseases that result from them. In the treatment of immune-related illnesses, developments in precision medicine—where treatments are customized based on a patient’s genetic and molecular profile—are showing promise. Highly targeted therapies have become possible due to their capacity to target particular molecules involved in immune responses, such as immunological checkpoints or miRNAs.

Additionally, modifying immune regulatory systems could result in less harmful and more effective treatments, improving the general standard of care for individuals with long-term illnesses. This is especially important in conditions like autoimmune illnesses, when immunological tolerance is compromised, and cancer, where immune evasion is a major factor in tumor growth. Promising results may be obtained from novel therapeutic approaches that try to improve or restore immune function. For the development of next-generation medicines, especially for diseases like cancer, autoimmunity, and chronic inflammatory disorders, further investigation into the molecular basis of immune control will be essential. In the end, these developments could lead to better patient outcomes, more individualized treatment plans, and more long-term strategies for treating immunological dysfunctions. Immunomodulation treatments have a promising future ahead of them, with increasing potential to revolutionize the management and treatment of immune-related illnesses.

## Figures and Tables

**Table 1:** miRNAs involved in immune regulation.

miRNA	Role in Immune Regulation	Target(s)	Acts on Cell Type(s)	Pathway/Function	References
miR-146a	Suppresses inflammation via NF-κB signaling	TRAF6, IRAK1	Macrophages, T-cells	Negative feedback on inflammation	[5,43]
miR-155	Promotes inflammation by enhancing cytokine production	SOCS1, SHIP1	T-cells, B-cells	Enhances Th1/Th17 responses	[5,43]
miR-21	Regulates T-cell activation and promotes Treg development	PTEN, PDCD4	Tregs	Facilitates immune tolerance	[55,56]
miR-223	Controls macrophage polarization	NFI-A, STAT3	Macrophages	Balances pro/anti-inflammatory phenotypes	[57,58]
miR-181	Enhances T-cell receptor sensitivity	Multiple phosphatases	T-cells	Modulates TCR signaling threshold	[59]
miR-125b	Modulates proinflammatory cytokines	TNF, IL-6	Macrophages, T-cells	Downregulates cytokine storms	[60]
miR-150	Regulates differentiation of B and T cells	c-Myb	B-cells, T-cells	Promotes memory B-cell and Treg development	[61,62]
miR-10a	Promotes Treg stability and suppressive function	Bcl-6, NF-κB	Tregs	Maintains immune homeostasis	[63]
miR-29a	Suppresses Th1 responses and IFN-γ production	T-bet, Eomes	CD4+ T-cells	Balances Th1/Th2 responses	[64]
miR-27a	Enhances Th17 differentiation by targeting inhibitors of IL-6 signaling	Runx1, Foxp3	CD4+ T-cells (Th17)	Facilitates inflammatory autoimmunity	[65]
miR-31	Promotes Th1 and Th17 differentiation while limiting Treg development	RhoA	T-cells	Enhances inflammatory response	[66]
miR-326	Enhances Th17 cell differentiation	Ets-1	Th17 cells	Drives autoimmune responses	[67]
miR-150-5p	Modulates natural killer cell activity	Myb	NK cells	Regulates NK cytotoxicity	[68]
miR-26a	Regulates TGF-β signaling and Treg homeostasis	SMAD1, SMAD4	Tregs	Promotes immune tolerance	[69]
miR-124	Reduces microglial activation and neuroinflammation	STAT3, C/EBP-α	Microglia	Suppresses neuroinflammatory diseases	[70]
miR-34a	Induces apoptosis and modulates macrophage polarization	Bcl-2, SIRT1	Macrophages	Enhances antitumor immunity	[71]
miR-15b	Regulates cytokine production and T-cell apoptosis	BCL2, STAT3	T-cells, B-cells	Modulates immune responses in infections	[72]
miR-221	Suppresses T-cell proliferation and migration	p27kip1	CD8+ T-cells	Restricts cytotoxic T-cell overactivation	[73]
miR-146b	Enhances Treg suppressive capacity	TRAF6, STAT1	Tregs	Reinforces NF-κB inhibition	[74]
miR-106a	Inhibits Th1 differentiation and cytokine secretion	IL-10, STAT3	T-cells	Promotes Th2 skewing	[75]
miR-142	Modulates dendritic cell maturation and Treg differentiation	APC gene family	Dendritic cells, Tregs	Coordinates antigen presentation	[76]
miR-181c	Controls mitochondrial metabolism in activated T-cells	COX1, COX2	T-cells	Maintains energy balance during activation	[77]
miR-222	Balances macrophage M1/M2 polarization	NF-κB, STAT6	Macrophages	Regulates tissue repair and inflammation	[78]

**Table 2:** Summary of key immune regulatory mechanisms.

No.	Mechanism	Gene(s) Involved	Regulators (Effect)	Acts on Cell Type(s)	Pathway/Function	References
1	Cytokine Signaling	IL6, IL10, TGFB1	IL-6 (pro-inflammatory), IL-10 (anti-inflammatory), TGF-β (anti-inflammatory)	T-cells, macrophages	Balances inflammation and immune suppression	[18,19,20]
2	Immune Checkpoints	PDCD1, CTLA4	PD-1 (inhibits T-cell activation), CTLA-4 (inhibits co-stimulation)	T-cells, APCs	Maintains self-tolerance and prevents autoimmunity	[23,24,25,26,27,28]
3	Regulatory T Cells (Tregs)	FOXP3, IL2RA	FoxP3 (essential for Treg development), TGF-β (induces Tregs), IL-2 (promotes Treg survival)	Tregs	Suppresses immune responses and promotes tolerance	[12,13,14]
4	Suppressor T Cells	IL10, TGFB1, CTLA4	IL-10 (anti-inflammatory), TGF-β (anti-inflammatory), CTLA-4 (blocks costimulation)	Tr1 cells, Tregs	Inhibits effector T-cell activation and inflammatory responses	[15,16,17]
5	Epigenetic Modifications	HDAC9, DNMT3A	Histone acetylation (activates genes), DNA methylation (silences genes)	T-cells, macrophages	Regulates immune cell differentiation and function	[37,38,39,40,41,42]
5a	Non-Coding RNAs	MIR146A, MIR155	miR-146a (anti-inflammatory), miR-155 (pro-inflammatory)	T-cells, B-cells, macrophages	Modulates cytokine signaling, TCR activation, and inflammation	[43,44,45]
6	Immunometabolism	MTOR, PRKAA1	mTOR (promotes effector T-cell activation), AMPK (enhances Treg activity)	Tregs, effector T-cells, macrophages	Links metabolic states to immune cell function	[46,47,48,49]
7	Apoptosis	BCL2, FAS	Bcl-2 (anti-apoptotic), Fas (pro-apoptotic)	T-cells, B-cells	Eliminates autoreactive or excess immune cells	[50,51,52]
8	Antigen Presentation	HLA-DRA, CD74	MHC-II (presents antigens), CD74 (regulates antigen loading)	Dendritic cells, macrophages	Initiates adaptive immune responses	[53,54,55]
9	Oxidative Stress	NOX2, SOD2	NOX2 (pro-oxidative), SOD2 (antioxidative)	Macrophages, neutrophils	Modulates inflammation and pathogen elimination	[56,57,58]
10	Autophagy	ATG5, ATG7	Beclin-1 (pro-autophagic), mTOR (inhibits autophagy)	T-cells, macrophages	Maintains immune homeostasis and antigen presentation	[59,60,61]
11	Complement System	C3, C5AR1	C3 (pro-inflammatory), C5a receptor (enhances inflammation)	Neutrophils, macrophages	Bridges innate and adaptive immunity	[62,63,64]

**Table 3:** Health conditions associated with dysfunction in immune regulation processes.

No.	Mechanism	Defect Type	Associated Disease(s)/Condition(s)	Pathophysiology/Impact	References
1	Cytokine Signaling	Elevated IL-6	Rheumatoid arthritis, cytokine storm	Promotes excessive inflammation, tissue damage, and autoimmunity	[32,33]
2	Immune Checkpoints	PD-1/CTLA-4 inhibition	Cancer, autoimmune disorders	Failure to inhibit T-cell activity leads to autoimmunity; inhibition helps tumor evasion	[34,35,36]
3	Regulatory T Cells (Tregs)	FOXP3 mutation (loss of function)	Autoimmune polyendocrinopathy, IPEX syndrome	Impaired Treg development causes unregulated effector T-cell responses	[28,29]
4	Suppressor T Cells	Low IL-10/TGF-β levels	Chronic inflammatory diseases, Crohn’s disease	Reduced suppression of inflammatory responses leads to persistent inflammation	[30,31]
5	Epigenetic Modifications	Aberrant DNA methylation patterns	Multiple sclerosis, systemic lupus erythematosus	Dysregulated gene expression disrupts immune tolerance and promotes autoimmunity	[37,38,39]
6	Non-Coding RNAs	Dysregulated miRNA expression	Autoimmune diseases, inflammatory conditions	Misregulation of miRNAs alters cytokine signaling and immune cell activation	[43,44,45]
7	Immunometabolism	mTOR hyperactivity	Obesity, metabolic syndrome, type 2 diabetes	Increased effector T-cell activation and reduced Treg function worsen metabolic inflammation	[46,48,49]
8	Oxidative Stress	Excessive ROS production	Atherosclerosis, chronic obstructive pulmonary disease (COPD)	Oxidative stress damages tissues and promotes chronic inflammation	[50,51,52]
9	Autophagy	Impaired autophagy pathway	Neurodegenerative diseases, Crohn’s disease	Reduces clearance of pathogens and cellular debris, increasing inflammatory responses	[53,54,55]
10	Complement System	Uncontrolled complement activation	Paroxysmal nocturnal hemoglobinuria, lupus	Overactivation amplifies tissue injury and autoantibody-mediated damage	[56,57,58]

## Data Availability

All data are available from the text or can be passed on by the author upon request.
